# Information meetings on end-of-life care for older people by the general practitioner to stimulate advance care planning: a pre-post evaluation study

**DOI:** 10.1186/s12875-021-01463-3

**Published:** 2021-06-07

**Authors:** Annicka G. M. van der Plas, H. Roeline W. Pasman, Roosmarijne M. K. Kox, Marianne Ponstein, Bea Dame, Bregje D. Onwuteaka-Philipsen

**Affiliations:** 1 Department of Public and Occupational Health, Amsterdam Public Health Research Institute, Amsterdam UMC, Location VU University Medical Center, PO Box 7057, 1007 MB Amsterdam, the Netherlands; 2 Zorggroep Almere, Almere, The Netherlands

**Keywords:** Advance care planning, Advance directives, Communication, General practice, Health services for the aged, Physician–patient relations

## Abstract

**Background:**

To increase knowledge about options people have concerning end-of-life-care issues, General Practitioners (GPs) can organise meetings to inform their older patients. We evaluated these meetings, using the following research questions: How did the attendees experience the information meeting? Was there a rise in Advance Care Planning (ACP) behaviour after the information meeting? Was there a change in trust people have that physicians will provide good care at the end of life and that they will follow their end-of-life wishes after the information meetings?

**Methods:**

Four GPs invited all patients of 75 years and older registered in their GP practices to the meeting via a written letter. Four meetings of 2 h took place in 2016. Meetings started with a presentation on end-of-life topics and ACP by the GP followed by time for questions.

A pre-post evaluation study was done using written questionnaires distributed and filled in at the start of the meeting (T_0_) at the end of the meeting (T_1_) and 6 months after the meeting (T_2_).

**Results:**

In total 225 older people attended a meeting of which 154 (68%) filled in the questionnaire at T_0_ and 145 (64%) filled in the questionnaire at T_1_. After six months, 90 of the 121 people who approved of being sent another questionnaire at T_2_, returned it (40%). The average age of the respondents was 80 years (T_0_). The meetings were evaluated positively by the attendees (T_1_). ACP issues (appointing a proxy, resuscitation, hospitalisation, euthanasia, treatment preferences under certain circumstances, preferred place of care and nursing home admittance) were discussed with a physician, a relative or both more often in the 6 months after having attended the meeting (T_2_), compared to before (T_0_). Compared to before the meeting (T_0_), trust in the GP providing good end-of-life care and following end-of-life wishes was higher immediately after the meeting (T1), but not after 6 months (T_2_).

**Conclusion:**

Information meetings on end-of-life care by GPs have a positive influence on the occurrence of ACP, both with the physician and others. Although, this method especially reaches the older people that are already interested in the subject, this seems a relatively easy way to stimulate ACP.

**Supplementary Information:**

The online version contains supplementary material available at 10.1186/s12875-021-01463-3.

## Background

Many people would like to be cared for at home at the end of life [1, 2]. In the Netherlands, the general practitioner (GP) plays a central role in end-of-life care for people in the community. Almost all Dutch residents are registered with a GP, who functions as a gatekeeper for more specialised forms of care [3]. Therefore, it is important for GPs to know the care preferences of their patients, especially as people grow older and their chances of having to make choices about the care they do or do not want are likely to increase and may change over time.

Advance care planning (ACP) enables individuals to define goals and preferences for future medical treatment and care, to discuss this with their family and healthcare providers, and to record and update these preferences if necessary [4]. Although older people and their GPs are aware of the advantages of ACP [5], such conversations are not yet custom in GP practices [6]. The prevalence of Advance Directives and/or ACP conversations in the older population in general is low, with rates between 3.2% for people aged 65 years or older in primary care in Australia, and 12.6% for people aged 90 years or older admitted to an UK hospital with an emergency [7, 8]. In the Netherlands, 10% of the people aged 61 years or older have a living will [9]. In a recent study [10], a lack of trust or negative previous experiences with ACP could be a reason for older people not wanting to participate in ACP conversations; however, older people who did engage in ACP felt they could trust their GP more afterwards.

One barrier to engage in ACP often mentioned by the GP is time [11–13]. From the older person’s viewpoint a barrier is lack of knowledge about potential health care choices and options, but also awareness involving their personal norms and values [14–16]. One way to overcome these barriers is for GPs to organise information meetings for their patients. This could be a time efficient way to increase older people’s knowledge of options for care and treatment at the end of their life. What sets these meetings apart from other public information meetings, is that they are organised by the patients’ own GP.

Four GPs in the Netherlands took the initiative to organize information meetings for their older patient population, because they realized in their daily practices that older people often lacked basic information about palliative care and end-of-life issues. They asked researchers from a university to evaluate these information meetings.

In this paper we describe the evaluation of these meetings. The research questions were:

1. How did the attendees experience the information meeting?
2. Was there a rise in ACP behaviour after the information meeting?
3. Was there a change in trust people have that physicians will provide good care at the end of life and that they will follow their end-of-life wishes after the information meetings?

## Methods

### Design of the evaluation study

A pre- post evaluation study using written questionnaires distributed and filled in at the start of the meeting (T_0_), at the end of the meeting (T_1_) and 6 months after the meeting (T_2_) to evaluate the information meetings.

### Information meetings

The information meetings were held by GPs from four different group practices in the provinces Flevoland and Noord-Holland, and were held from April until August 2016. The participating GPs decided to target all patients of 75 years and older registered in their practices, as older people form a relevant group for ACP and they felt it important to limit the potential number of participants per meeting. Patients were invited by their own GP by a written letter; they could enrol themselves by returning a paper slip. They could come with a companion. Sometimes this was somebody, e.g. a partner of almost 75 or with a chronic illness, who themselves were also interested in the meeting. Other times it was somebody, for example a child, who only came along to accompany a participant.

Three information meetings were held in the GP practice building, one in a church (because the GP practice was too small). The meetings lasted about 2 h, started with a powerpoint presentation on end-of-life topics and ACP by the GP, followed by time for questions. After the first meeting, the presentation was adapted to provide more information on palliative care. In the following three meetings, the GPs in that meeting used the same adapted powerpoint presentation.

### Data collection

When arriving at the venue, before the meeting started, the researchers handed out the questionnaires for T_0_, and T_1_. Attendees were asked by the researchers to fill in the first questionnaire before the meeting started (T_0_). At the end of the meeting they were asked to fill in a second questionnaire (T_1_). The researchers collected the T_0_ and T_1_ questionnaires when the participants left the meeting.

Participants were also asked to fill in a consent form if they consented to the GP sending them a last questionnaire 6 months after the meeting (T_2_). If so, the GP sent the questionnaire after 6 months to the respondents home address and respondents could return the questionnaire to the researchers using a pre-addressed return envelope. With this procedure, the researchers did not have identifying data about the respondents, and GPs did not have the completed questionnaires. The questionnaires had a unique identification number to keep track of the response. Data were stored on a secured network drive of Amsterdam UMC that is only accessible to the researchers.

### Questionnaires

The questionnaire at the start of the meeting (T_0_) contained questions on expectations of the meeting and reasons for coming to the meeting, questions on health, illness and quality of life, trust in the GP, previous experience with ACP, and demographics. The questionnaire at the end of the meeting (T_1_) contained questions evaluating the meeting, ACP related plans after the meeting, and trust in the GP. The questionnaire 6 months after the meeting (T_2_) had questions on health, illness and quality of life, trust in the GP, experience with ACP, and demographics. All questionnaires were specifically developed for this study. See the additional file for the questionnaires.

### Analyses

**Fig. 1:**
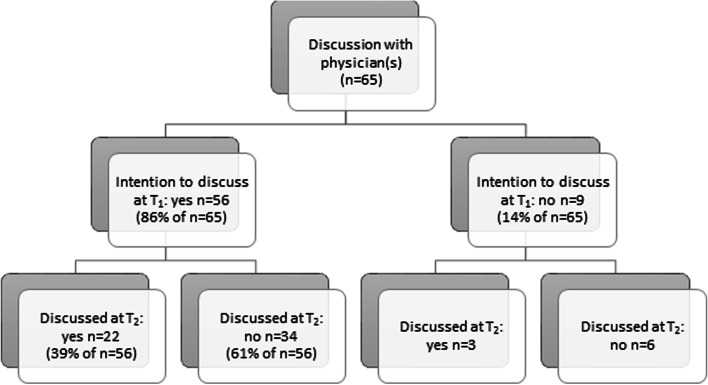
Plans to have a conversation with their physicians and execution of plans 6 months later^1^. ^1^Selection of attendees who filled in T_1_ and T_2_ (*n* = 86). Missing data: *n* = 21

**Fig. 2:**
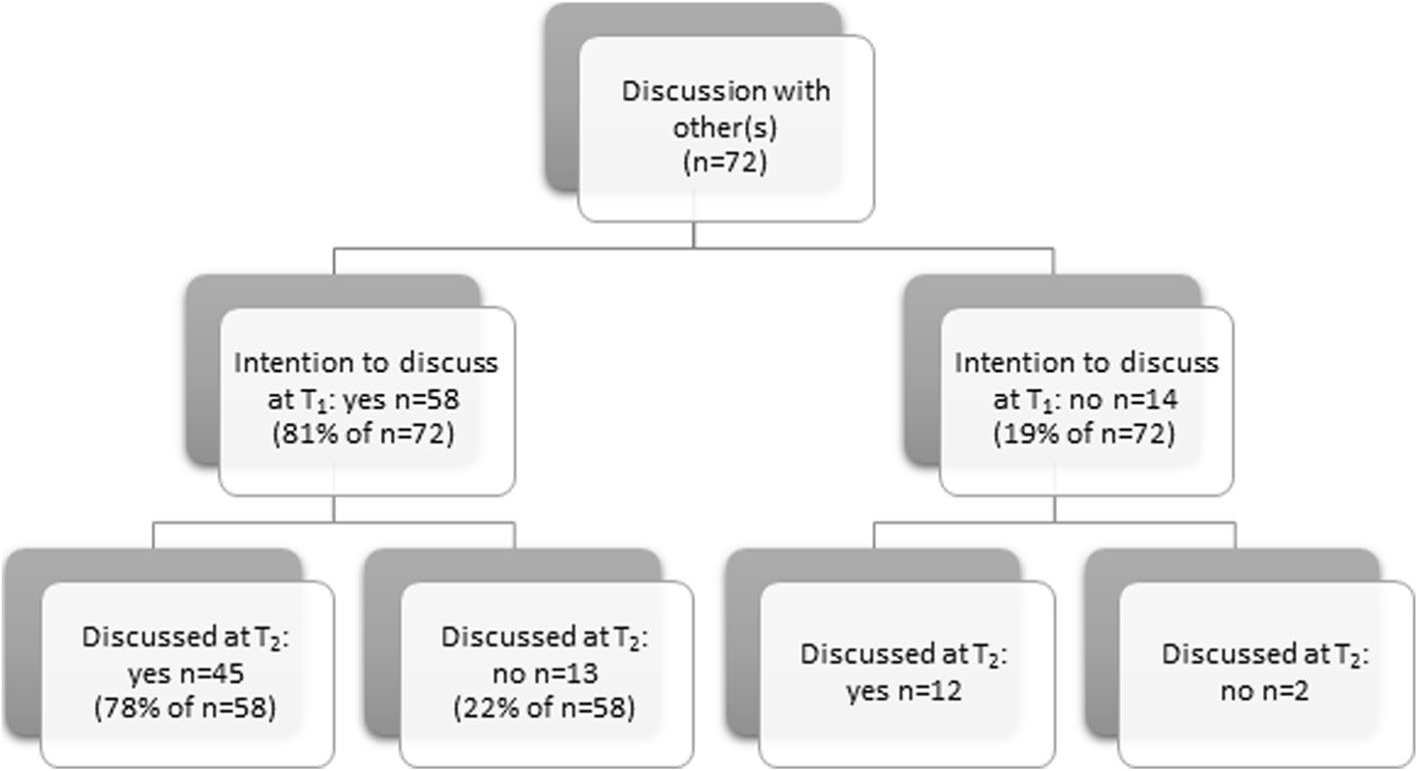
Plans to have a conversation with others and execution of plans 6 months later. Selection of attendees who filled in T_1_ and T_2_ (*n* = 86). Missing data: *n* = 14

**Fig. 3:**
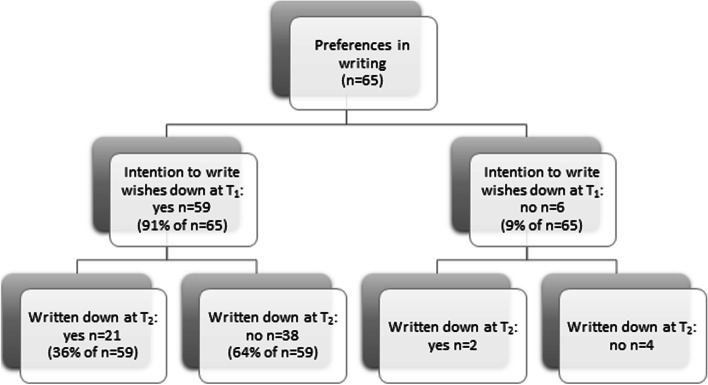
Plans to write wishes down and execution of that plan 6 months later. Selection of attendees who filled in T_1_ and T_2_ (*n* = 86). Missing data: *n* = 21

Descriptive statistics were used to summarise the characteristics of attendees. Answers to the open questions on expectations and the evaluation of the meeting were categorised. Experiences with the meeting were analysed using the Crosstabs procedure with the Pearson Chi-Square test. Pre- and post-test differences in trust were tested with a non-parametric test for paired samples (marginal homogeneity test). Because of small percentages in the categories ‘not much trust’ and ‘no trust’ these were grouped together. Pre- and post-test differences in ACP conversations and Advance Directives were analysed in McNemar-test for paired samples performed on attendees who filled in a questionnaire before and 6 months after the information meeting. In Figs. 1, 2 and 3 the questions on plans regarding talking to a physician, others and writing wishes down at T_1_ were combined with whether or not an ACP conversation with the physicians or others had taken place or whether preferences were written down at T_2_. This was derived from the answers on the seven ACP topics. If the answer for at least one of the seven topics was ‘I have discussed this with a physician’ than it was concluded that an ACP conversation with the physician had taken place. The same was done for talking to others and putting wishes down in writing. We did not impute missing observations. Statistical analyses were done with SPSS version 20.

## Results

### Participants

In total 225 older people attended a meeting (Table 1) of which 154 (68%) filled in the questionnaire at T_0_ and 145 (64%) filled in the questionnaire at T_1_. After six months, 90 of the 121 people who approved of being sent another questionnaire at T_2_, returned it (40%).

**Table 1:** Participation in information meetings and response on T_0,_ T_1_ and T_2_ for the 4 information meetings (abs. numbers and percentage of all participants at meeting)

	*Total*	*Meeting A*	*Meeting B*	*Meeting C*	*Meeting D*
	*n (%)*	*n (%)*	*n (%)*	*n (%)*	*n (%)*
*Participants at the meeting*^*a*^	*225*	*35*	*80*	*65*	*45*
*Filled out questionnaire at the start of the meeting (T*_*0*_*)*	*154 (68%)*	*31 (89%)*	*51 (64%)*	*42 (65%)*	*31 (69%)*
*Filled out questionnaire at the end of the meeting (T*_*1*_*)*	*100 (64%)*	*29 (83%)*	*51 (64%)*	*38 (58%)*	*27 (60%)*
*Gave informed consent to send questionnaire after 6 months*	*121 (54%)*	*24 (69%)*	*46 (58%)*	*27 (42%)*	*24 (53)*
*Filled out questionnaire after 6 months (T*_*2*_*)*	*90 (40%)*	*19 (54%)*	*38 (48%)*	*19 (29%)*	*14 (31%)*

^a^excluding people that were only there to accompany a participant

The majority of attendees were female (61%) and the mean age of attendees was 80 years (Table 2). Although only people aged 75 years or older were invited, some attendees were younger, for instance because the invited person came with a partner or other companion. The majority perceived their health as good or very good (84%).

**Table 2:** Background characteristics as reported on measurement T_0_ of attendees of information meetings (April – August 2016) who filled in questionnaires T_0_ (*n* = 154), T_1_ (*n* = 145) and T_2_(*n* = 90)

	Before the start of the meeting^a^ *n* = 154^b^		Immediately after meeting *n* = 145^c^		6 Month after meeting *n* = 90^d^	
	n	%	n	%	n	%
*Female*	93	61	84	59	51	59
Age, mean (range)	79.6 (57–91)		79.5 (57–91)		79.4 (66–89)	
*Educational level*						
- Low	72	51	68	50	40	49
- Middle	36	25	35	26	18	22
- High	34	24	33	24	23	28
*Perceived health*						
- Very good	11	7	11	8	6	7
- Good	115	77	110	78	65	78
- Less than good	24	16	20	14	12	15
*Perceived quality of life*						
- Very good	30	20	29	20	18	21
- Good	107	71	100	70	61	73
- Less than good	13	9	13	9	5	6
*Diseases (self reported)*						
No	43	30	41	30	21	26
Yes, namely	102	70	95	70	60	74
- Cancer	11	8	9	7	6	7
- Rheumatism	28	19	26	19	20	25
- Lung disease	17	12	17	13	9	11
- Diabetes	31	21	27	20	18	22
- Heart disease	40	28	37	27	25	31
- (Consequences of) stroke	8	6	8	6	4	5
- Dementia	1	1	1	1	0	0
- Other	14	10	12	9	4	5

^a^ The background characteristics as reported in this table were all from measurement T_0_ (so be aware, that for example the reported health care status in column 3 is from 6 months earlier)

^b^ missing observations T_0_: sex 2; age 2; education 12; health status and quality of life 4; diseases 9

^c^ missing observations T_1_: sex 2; age 2; education 9; health status 4; quality of life 3; diseases 9

^d^ missing observations T_2_: sex 4; age 4; education 9; health status 7; quality of life 6; diseases 9

### Expectations of and experience with the information meeting

More than half of attendees attended the meeting because they wanted to know more about the end-of-life (57%) or because they think about the end-of-life (52%). Most attendees expected information on practices to hasten death (euthanasia, assisted suicide, voluntarily stopping eating and drinking (VSED) (28%) and possibilities of palliative care (21%). They were asked immediately after the meeting (T_1_) if the meeting matched their expectations, which was the case for a majority (63%) of participants. They could explain their answer regarding expectations in an open question. A total of 85 comments were given, which were categorised into 113 codes. Most (67%) answers were positive, for instance that the meeting was good or that they thought the information was clear. A total of 18 (9%) comments were less positive, mostly pertaining to inaudibility of the questions asked (one meeting was in a church and the acoustics were bad, in another meeting only one microphone was available which made interaction problematic) or lengthy elaborations of other attendees on personal matters during the discussion. The information on Advance Directives (21%) and the importance of talking to family, friends and care providers (15%) were mentioned most when asked about the most important thing they had heard.

The meetings were evaluated positively on the topics discussed (21% very good; 72% good), clarity of information (30% very good; 56% good), possibility to ask questions (29% very good; 68% good) and answers to questions posed (26% very good; 63% good). With regard to clarity of the information and answers to questions meeting B appeared to be evaluated more favourably, compared to meetings A, C and D (Supplementary Table A.1, Additional File 1).

### Advance care planning

Six months after the meeting attendees more often had thought about (68% vs 89%), discussed with physicians (5% vs 18%) or others (10% vs 36%) and written down (7% vs 18%) preferences regarding treatments under certain circumstances, compared to before (Table 3). Attendees more often thought about (63% vs 81%) hospitalisation and discussed this with another person (not a physician) (8% vs 29%) more often after the meeting compared to before.

**Table 3:** Advance Care Planning before and 6 months after the information meeting

	Before the meeting^a^ *N* = 86	6 months after^a^ *N* = 86	*p*-value**
	n (%)	n (%)	
*Who should decide for me when I cannot do it my self*			
- Thought about it	73 (88%)	80 (93%)	**0.031**
- Discussed with physician	7 (8%)	17 (20%)	**0.008**
- Discussed with another person	19 (23%)	44 (52%)	** < .001**
- Written down	12 (15%)	20 (24%)	**0.016**
*Whether I want to be resuscitated*			
- Thought about it	69 (84%)	77 (94%)	**0.008**
- Discussed with physician	7 (9%)	18 (22%)	**0.001**
- Discussed with another person	18 (22%)	39 (48%)	** < .001**
- Written down	7 (9%)	14 (17%)	**0.006**
*Which treatments I would or would not want under certain circumstances*			
- Thought about it	55 (68%)	71 (89%)	** < .001**
- Discussed with physician	4 (5%)	14 (18%)	**0.004**
- Discussed with another person	8 (10%)	29 (36%)	** < .001**
- Written down	6 (7%)	14 (18%)	**0.008**
*Whether or not I can / want to stay at home*			
- Thought about it	65 (79%)	69 (83%)	0.629
- Discussed with physician	0	3 (4%)	0.250
- Discussed with another person	16 (20%)	28 (34%)	**0.023**
- Written down	2 (2%)	5 (6%)	0.453
*Whether or not I would want to be admitted to hospital*			
- Thought about it	52 (63%)	66 (81%)	**0.004**
- Discussed with physician	0	4 (5%)	0.125
- Discussed with another person	7 (8%)	24 (29%)	** < .001**
- Written down	0	4 (5%)	0.125
*Whether or not I want to be admitted to a nursing home*			
- Thought about it	64 (78%)	69 (83%)	0.454
- Discussed with physician	0	5 (6%)	0.063
- Discussed with another person	12 (15%)	34 (41%)	** < .001**
- Written down	3 (4%)	5 (6%)	0.688
*Whether I would want euthanasia in certain circumstances*			
- Thought about it	74 (90%)	73 (92%)	0.722
- Discussed with physician	5 (6%)	15 (19%)	**0.022**
- Discussed with another person	20 (24%)	36 (46%)	**0.015**
- Written down	9 (11%)	13 (17%)	0.388

**Related Samples Mc Nemar Change Test

^a^ Selection of attendees who filled in both T_0_ and T_2_ (*n* = 86). Missing values: who should make decisions for me T_0_*n* = 3 T_2_*n* = 3; resuscitation T_0_*n* = 4 T_2_*n* = 4; treatments T_0_*n* = 5 T_2_*n* = 6; staying at home T_0_*n* = 4 T_2_*n* = 3; hospital T_0_*n* = 3 T_2_*n* = 4; nursing home T_0_*n* = 4 T_2_*n* = 3; euthanasia T_0_*n* = 4 T_2_*n* = 7

Figures 1  and 3 show that attendees who plan to speak to their physicians (*n* = 56) or plan to put down their wishes in writing (*n* = 59) have done so six months later in 39% (*n* = 22) and 36% (*n* = 21) of the cases. Attendees with plans to discuss wishes with others (*n* = 58) have acted accordingly in 78% (*n* = 45) of the cases, and a further 12 out of 14 attendees who did not initially plan to do so did discuss their preferences with others (Fig. 2).

### Trust in physicians to provide good end of life care and to follow end-of-life wishes

Attendees trusted the physicians more to provide good care at the end of life (p-value 0.002) and to follow end-of-life wishes (p-value < 0.001) after the meeting, compared to before (Table 4). This difference disappeared after six months. Posthoc analyses showed that attendees who had spoken to a physician after six months scored higher on trust to provide good care (p-value 0.003) and to follow end-of-life wishes (p-value 0.002) six months after the meeting, compared to attendees who had not spoken to a physician.

**Table 4:** Trust in physicians before, immediately after and 6 months after the information meeting

	Before the information meeting (T_0_) *N* = 154	Immediately after (T_1_)^a^ *N* = 144	Difference T_0_ – T_1_, *p*-value**	6 months after (T_2_)^a^ *N* = 86	Difference T_0_ – T_2_, *p*-value**
	n (%)	n (%)		n (%)	
*How much do you trust physicians to provide good care to you in the final stage of life?*^b^			**0.002**		0.465
- Very much trust	47 (32)	61 (45)		28 (34)	
- Reasonably much trust	82 (57)	64 (47)		48 (58)	
- Not much or no trust	16 (11)	12 (9)		7 (8)	
*How much do you trust physicians to follow your wishes about medical decisions at the end of your life?*^c^			** < 0.001**		0.869
- Very much trust	44 (30)	51 (37)		19 (24)	
- Reasonably much trust	79 (55)	79 (57)		53 (66)	
- Not much or no trust	22 (15)	8 (6)		8 (10)	

^**^ marginal homogeneity test for paired samples

^a^ selection of people who also filled in the questionnaire before the information meeting; there was one person who did fill in T_1_ but not T_0_, there were 4 persons who did fill in T_2_ but not T_0_

^b^ missing observations: between 9 and 3 per group

^c^ missing observations between 9 and 6 per group

## Discussion

The information meetings were evaluated positively by the attendees and yielded positive outcomes. Six months after the meeting attendees more often had thought about, discussed with physicians or others and written down preferences regarding appointing a proxy, resuscitation, and treatment preferences under certain circumstances, compared to before. Attendees more often had thought about hospitalisation and discussed this with another person (not a physician) after the meeting than before. Whether the attendee could or wanted to stay at home and nursing home admittance were discussed with another person more often after the meeting, compared to before. Euthanasia was discussed with a physician and another person more often after the meeting, compared to before. The percentage of attendees who have acted on plans to discuss wishes with others was higher (78%) than the percentage of attendees who have acted on plans to discuss wishes with physicians (39%) and put preferences in writing (36%). Trust was higher immediately after the meeting, but not after 6 months.

The meetings seem to be a time efficient way to stimulate ACP conversations with the GP and others. A recent study on two public health interventions (‘Awareness-Raising’ presentations and ‘How to’ workshops) also yielded positive results with regard to stimulating discussions [17]. The GPs did not receive a remuneration for organising these meetings. The GPs gave the presentation themselves and used their own staff in supporting functions (such as sending the invitations), so they invested their own time but did not need to pay for a guest speaker and external staff. The presentation was ‘recycled’, as was the road map to organise the meeting, so other GPs did not have to make these themselves. Also, the locations were free or inexpensive (e.g. because of a discount rate for social organisations). Per meeting 35 to 80 people participated and thus received information about the options they have concerning end-of-life-care issues. Our next step is to implement these meetings in other GP practices and compare meetings by GPs with meetings organised by social organisations. Our hypothesis is that meetings organised by GPs may draw a broader audience (people who have not thought about end-of-life very much yet) because they are enticed by receiving a personal letter from their own GP (instead of seeing a notice in a paper or a flyer in the supermarket).

The data suggest it may be easier to realise plans to discuss wishes with others, compared to talking to physicians or putting preferences in writing. Previous research also indicated that conversations more often were with family or friends than with a physician [18]. Talking with family or friends involves a lot less organisation, especially if people see each other regularly and the topic may come up spontaneously. For a conversation with the physician an appointment must be made and to put wishes into writing some preparation is involved (e.g. looking up advance directives on the internet) and some thought has to be put into the wording of the document. Without a sense of urgency these actions may easily be postponed [19, 20]. However, from the point of view from the GP, the attendance list of the meeting in itself may provide valuable information. A recent publication showed that GPs make a selection in people they have conversations with [21]. If GPS take readiness to engage in ACP into account when they take the initiative to start a conversation, people who attended the meeting would be a good starting point.

Immediately after the meetings, trust in physicians to provide good care and to follow wishes was higher than before and six months after the meeting. This may indicate that attendees felt that the GP came across as knowledgeable or nuanced (e.g. made distinctions in personal circumstances that could influence preferences) and/or felt that the role of the GP in care delivery and decision making was clearly discussed. The effect disappeared after 6 months. However, when comparing attendees who had or had not spoken to a physician after six months, those who had scored higher on trust than those who had not spoken to a physician. Future studies should explore this rise and fall of trust and the relation with ACP more deeply. Other studies indicate a complex interaction between trust and ACP [10, 13–15, 22].

### Strengths and limitations of this study

There are few studies on information meetings [17, 23], and we did not find previous studies of end-of-life information meetings initiated by GPs. The information meetings were held by GPs from four different group practices. Participating GPs were self-selected; they felt palliative care is important. GPs in general may be less inclined to organise meetings, and may be less capable to give the presentation and answer questions themselves. Also, we did not have information on how many people received an information letter and therefore could not study what percentage of invited people took up the invitation and attended the meeting. Attendees may have been more interested in palliative care. Those less comfortable with the topic were probably less likely to accept the invitation, resulting in a selection bias for the study and a diminished reach of the meetings. Future studies should explore strategies to help bring a broader selection of patients to the meetings.

## Conclusion

The meetings seem to be a time efficient way to stimulate ACP discussions with the GP and others. The meetings were evaluated positively by the attendees and ACP discussions increased after the meetings, especially with friends and family.

## Supplementary Information

**Additional file 1:** Questionnaire at T_0_. Questionnaire at T_1_. Questionnaire at T_2_. **Table A.1.** Experiences of 145 attendees with information meetings (April – August 2016).

## Data Availability

The data underlying this article will be shared on reasonable request to the corresponding author.

## Abbreviations

ACP: Advance care planning
GP: General practitioner
