# Supplementary material for: Information meetings on end-of-life care for older people by the general practitioner to stimulate advance care planning: a pre-post evaluation study
Source: BMC Fam Pract. 2021 Jun 7;22:109. doi: 10.1186/s12875-021-01463-3 (PMC8183039; doi:10.1186/s12875-021-01463-3)
Supplement: Supplementary file 1 — Additional file 1: Questionnaire at T0. Questionnaire at T1. Questionnaire at T2. Table A.1. Experiences of 145 attendees with information meetings (April – August 2016). [file 12875_2021_1463_MOESM1_ESM.docx]

**Information meetings on end-of-life care for older people by the General Practitioner to stimulate Advance Care Planning: a pre-post evaluation study**

**Additional file / Appendix**

Annicka G.M. van der Plas ^1^; H Roeline W Pasman ^1^; Roosmarijne M.K. Kox ^1^; Marianne Ponstein ^2^; Bea Dame ^2^; Bregje D. Onwuteaka-Philipsen ^1^

^1^ Amsterdam UMC, location VU University Medical Center, Department of Public and Occupational Health, Amsterdam Public Health research institute, Amsterdam, The Netherlands

^2^ Zorggroep Almere, Almere, The Netherlands

Corresponding author: dr. A.G.M. van der Plas. Amsterdam UMC, location VU University Medical Center, Department of Public and Occupational Health, PO Box 7057, 1007 MB Amsterdam, the Netherlands. E-mail: [eol@amsterdamumc.nl](mailto:eol@amsterdamumc.nl)

**Questionnaire at T_0_**

1) What is your reason to attend this meeting? (more than one answer possible)

❑ Because the subject is relevant to me

❑Because I want to know more about the end of life

❑ Because I think about the end of life

❑ Other people said I should go

❑ Because …………………………………………………….

2) What do you think off with regard to preferences at the end of life? What topics come to mind?

* ………………………………………………………………………………………

* ……………………………………………………………………………………….

* ……………………………………………………………………………………….

* ……………………………………………………………………………………….

3) What information do you expect to receive in this meeting?

…………………………………………………………………………………………

………………………………………………………………………………………….

………………………………………………………………………………………….

4) What is your age?: _____ years

5) You are: male female

6) What is the highest education you received?:______________________

7) In general, what is your state of health?

❑ Very good

❑ Good

❑ Less than good

8) In general, how is your quality of life?

❑ Very good

❑ Good

❑ Less than good

9) Do you have one or more of the following conditions? (more than one answer possible)

❑ No

❑ Yes, namely:

*❑ cancer*

*❑ rheumatism*

*❑ lung disease*

*❑ diabetes*

*❑ heart disease*

*❑ (consequences of) stroke*

*❑ Dementia*

*❑ Multiple Sclerose (MS)*

*❑ Amyotrofische Lateraal Sclerose (ALS)*

*❑ Depression*

*❑ Anders, namely*

10) *How much do you trust your physicians to provide good care to you in the final stage of life?*

❑ Very much trust

❑ Fairly much trust

❑ Not much trust

❑ No trust

11) How much do you trust your physicians to follow your wishes concerning medical decisions at the end of your life?

❑ Very much trust

❑ Fairly much trust

❑ Not much trust

❑ No trust

12) Have you thought and/or talked and/or written down your wishes about… (*per topic* more than one answer possible)

|  | not thought about it | thought about it | talked to a physician | talked to someone else | ) I have recorded it |
| --- | --- | --- | --- | --- | --- |
| Who should decide for you if you cannot do that yourself? |  |  |  |  |  |
| Whether you can and want to stay at home? |  |  |  |  |  |
| Whether or not you would want to go to the hospital? |  |  |  |  |  |
| Whether or not you would want to be admitted to a nursing home? |  |  |  |  |  |
| Whether you want to be resuscitated? |  |  |  |  |  |
| Which treatments you would and would not want to receive in certain circumstances? |  |  |  |  |  |
| Whether you would want euthanasia in certain circumstances? |  |  |  |  |  |
| Other topics?  …………………………………………….. |  |  |  |  |  |
| Other topics?  …………………………………………… |  |  |  |  |  |

13) Are there any other topics that you thought about, have spoken about, and/or have recorded your wishes?

* ………………………………………………………………………………………

* ……………………………………………………………………………………….

* ……………………………………………………………………………………….

* ……………………………………………………………………………………….

**Questionnaire at T_1_**

1) Did the meeting match your expectations?

❑ yes

❑ a bit

❑ no

Can you clarify this?

…………………………………………………………………………………………

………………………………………………………………………………………….

………………………………………………………………………………………….

2) How do you rate the meeting on the following aspects?

|  | Very bad | bad | not good/ not bad | good | very good |
| --- | --- | --- | --- | --- | --- |
| topics discussed | □ | □ | □ | □ | □ |
| clarity of information | □ | □ | □ | □ | □ |
| possibility to ask questions | □ | □ | □ | □ | □ |
| answers to questions posed | □ | □ | □ | □ | □ |

3) What is the most important thing you heard in this meeting?

……………………………………………………………………………..…………

………………………………………………………………………………………...

………………………………………………………………………………………..

4) After this meeting I intent to…

| 1. look for additional information | ❑ No  ❑ Yes, on: ……………………………….. |
| --- | --- |
| 1. discus wishes with family and/or friends | ❑ No  ❑ Yes, about: ……………………………….. |
| 1. discus wishes with my physician(s) | ❑ No  ❑ Yes, about: ……………………………….. |
| 1. record my wishes | ❑ No  ❑ Yes, about: ……………………………….. |

5) *How much do you trust your physicians to provide good care to you in the final stage of life?*

❑ Very much trust

❑ Fairly much trust

❑ Not much trust

❑ No trust

6) How much do you trust your physicians to follow your wishes concerning medical decisions at the end of your life?

❑ Very much trust

❑ Fairly much trust

❑ Not much trust

❑ No trust

7) Do you have suggestions to improve the meeting?

* ………………………………………………………………………………………

* ……………………………………………………………………………………….

* ……………………………………………………………………………………….

* ……………………………………………………………………………………….

**Questionnaire at T_2_**

1) What is your age?: _____ years

2) You are: male female

3) After the meeting on end-of-life care, did you discus the end-of-life with your GP?

❑ no

❑ yes, once

❑ yes, several times

Can you clarify this?

……………………………………………………………………………………………

……………………………………………………………………………………………

4) In general, what is your state of health?

❑ Very good

❑ Good

❑ Less than good

5) How is your current state of health, compared to your state of health six months ago (spring 2016)?

❑ Much better

❑ A bit better

❑ No difference

❑ A bit worse

❑ Much worse

6) In general, how is your quality of life?

❑ Very good

❑ Good

❑ Less than good

7) What do you think off with regard to preferences at the end of life? What topics come to mind?

* ………………………………………………………………………………………

* ……………………………………………………………………………………….

* ……………………………………………………………………………………….

* ……………………………………………………………………………………….

8) *How much do you trust your physicians to provide good care to you in the final stage of life?*

❑ Very much trust

❑ Fairly much trust

❑ Not much trust

❑ No trust

9) How much do you trust your physicians to follow your wishes concerning medical decisions at the end of your life?

❑ Very much trust

❑ Fairly much trust

❑ Not much trust

❑ No trust

10) Have you thought and/or talked and/or written down your wishes about… (*per topic* more than one answer possible)

|  | not thought about it | thought about it | talked to a physician | talked to someone else | ) I have recorded it |
| --- | --- | --- | --- | --- | --- |
| Who should decide for you if you cannot do that yourself? |  |  |  |  |  |
| Whether you can and want to stay at home? |  |  |  |  |  |
| Whether or not you would want to go to the hospital? |  |  |  |  |  |
| Whether or not you would want to be admitted to a nursing home? |  |  |  |  |  |
| Whether you want to be resuscitated? |  |  |  |  |  |
| Which treatments you would and would not want to receive in certain circumstances? |  |  |  |  |  |
| Whether you would want euthanasia in certain circumstances? |  |  |  |  |  |
| Other topics?  …………………………………………….. |  |  |  |  |  |
| Other topics?  …………………………………………… |  |  |  |  |  |

11) Are there any other topics that you thought about, have spoken about, and/or have recorded your wishes?

* ………………………………………………………………………………………

* ……………………………………………………………………………………….

* ……………………………………………………………………………………….

Table A.1. Experiences of 145 attendees with information meetings (April – August 2016)

|  | Total (T_1_)  N=145 | Meeting A (T_1_)  n=29* | Meeting B (T_1_)  n=51* | Meeting C (T_1_)  n=38* | Meeting D (T_1_)  n=27* | p-value** |
| --- | --- | --- | --- | --- | --- | --- |
|  | n (%) | n (%) | n (%) | n (%) | n (%) |  |
| Evaluation of the meeting with regard to the topics discussed |  |  |  |  |  | 0.428 |
| - very good | 29 (21%) | 5 (17%) | 16 (33%) | 4 (11%) | 4 (15%) |  |
| - good | 100 (72%) | 22 (76%) | 29 (59%) | 29 (83%) | 20 (77%) |  |
| - not good, not bad | 9 (7%) | 2 (7%) | 3 (6%) | 2 (6%) | 2 (8%) |  |
| - bad | 1 (1) | 0 | 1 (2%) | 0 | 0 |  |
| - very bad | 0 | 0 | 0 | 0 | 0 |  |
| Evaluation of the meeting with regard to clarity of the information |  |  |  |  |  | 0.019 |
| - very good | 41 (30%) | 5 (18%) | 23 (49%) | 5 (14%) | 8 (30%) |  |
| - good | 77 (56%) | 18 (64%) | 20 (43%) | 25 (69%) | 14 (52%) |  |
| - not good, not bad | 20 (15%) | 5 (18%) | 4 (9%) | 6 (17%) | 5 (19%) |  |
| - bad | 0 | 0 | 0 | 0 | 0 |  |
| - very bad | 0 | 0 | 0 | 0 | 0 |  |
| Evaluation of the meeting with regard to the possibility to ask questions |  |  |  |  |  | 0.236 |
| - very good | 40 (29%) | 6 (21%) | 21 (44%) | 7 (10%) | 6 (22%) |  |
| - good | 94 (68%) | 21 (75%) | 25 (52%) | 28 (80%) | 20 (74%) |  |
| - not good, not bad | 3 (2%) | 1 (4%) | 1 (2%) | 0 | 1 (4%) |  |
| - bad | 0 | 0 | 0 | 0 | 0 |  |
| - very bad | 1 (1%) | 0 | 1 (2%) | 0 | 0 |  |
| Evaluation of the meeting with regard to answers to the questions posed |  |  |  |  |  | 0.034 |
| - very good | 34 (26%) | 5 (18%) | 20 (43%) | 4 (13%) | 5 (20%) |  |
| - good | 83 (63%) | 17 (61%) | 25 (53%) | 24 (75%) | 17 (68%) |  |
| - not good, not bad | 14 (11%) | 6 (21%) | 1 (2%) | 4 (13%) | 3 (12%) |  |
| - bad | 1 (1%) | 0 | 1 (2%) | 0 | 0 |  |
| - very bad | 0 | 0 | 0 | 0 | 0 |  |

* missing data: topics discussed n=6; clarity of information n=7; possibility to ask questions n=7; answers to questions n=13.

** Pearson Chi-Square test
